# Effects of Acute Citrulline Malate Supplementation on CrossFit^®^ Exercise Performance: A Randomized, Double-Blind, Placebo-Controlled, Cross-Over Study

**DOI:** 10.3390/nu16193235

**Published:** 2024-09-24

**Authors:** Asli Devrim-Lanpir, Ferenc Ihász, Máté Demcsik, András Csaba Horváth, Pál Góczán, Péter Czepek, Johanna Takács, Rachel Kimble, Reza Zare, Fatma Esra Gunes, Beat Knechtle, Katja Weiss, Thomas Rosemann, Katie M. Heinrich

**Affiliations:** 1 School of Health and Human Performance, Dublin City University, D09 V209 Dublin, Ireland; asli.lanpir@dcu.ie; 2 Department of Nutrition and Dietetics, Faculty of Health Sciences, Istanbul Medeniyet University, 34862 Istanbul, Turkey; fatmaesra.gunes@medeniyet.edu.tr; 3 Faculty of Education and Psychology, Institute of Sport Sciences, Eötvös Lóránd University, 1075 Szombathely, Hungary; ihasz.ferenc@ppk.elte.hu (F.I.); demcsikmate@student.elte.hu (M.D.); handris99@student.elte.hu (A.C.H.); pulvsdi@student.elte.hu (P.G.); peter.czepek@gmail.com (P.C.); 4 Department of Social Sciences, Faculty of Health Sciences, Semmelweis University, 1085 Budapest, Hungary; 5 Division of Sport, Exercise and Health, School of Health and Life Sciences, University of the West of Scotland, Blantyre G720LH, UK; rachel.kimble@uws.ac.uk; 6 Meshkat Sports Complex, Karaj 3149645179, Alborz Province, Iran; 7 Arses Sports Complex, Karaj 3149645179, Alborz Province, Iran; 8 Institute of Primary Care, University Hospital Zurich, 8091 Zurich, Switzerland; katja@weiss.co.com (K.W.); thomas.rosemann@usz.ch (T.R.); 9 Medbase St. Gallen Am Vadianplatz, 9000 St. Gallen, Switzerland; 10 Department of Kinesiology, Kansas State University, Manhattan, KS 66506, USA; kmhphd@ksu.edu; 11 Department of Research and Evaluation, The Phoenix, Manhattan, KS 66502, USA

**Keywords:** citrulline malate, CrossFit^®^, exercise performance, cardiovascular function, ergogenic aid, heart rate zones, post-exercise recovery

## Abstract

Given the increasing popularity of CrossFit^®^ as a high-intensity functional training program and the potential benefits of citrulline malate (CM) in enhancing exercise performance through its role as a precursor to L-arginine and nitric oxide production, this study aimed to investigate the acute effects of CM supplementation on CrossFit^®^ performance and cardiovascular function. Using a randomized, double-blind, placebo-controlled, cross-over design, 21 recreationally active participants (mean age 22.2 ± 2.6 years, mean body weight 75.9 ± 10.4 kg) with CrossFit^®^ experience completed the “Cindy” workout under CM and placebo conditions. Participants consumed 4.4 g of CM or a placebo 60 min before the workout, and the performance was measured by the number of rounds completed. Secondary outcomes included heart rate response, time spent in different heart rate intensity zones, and post-exercise recovery time. The results indicated no significant difference in the number of rounds completed between the CM and placebo conditions (13.5 ± 5.2 vs. 13.8 ± 6.7 rounds, respectively; *p* = 0.587). However, the time spent in zone 4 (80–90% of HR max) was significantly increased in the CM condition (527 ± 395 s vs. 453 ± 334 s; *p* = 0.017), suggesting a potential benefit for aerobic capacity and anaerobic threshold. No significant differences in post-exercise recovery time were observed (6.6 ± 4.7 h vs. 6.9 ± 4.7 h; *p* = 0.475). This study highlights the need for further research with larger sample sizes, both genders, and different CM dosages to clarify these findings and better understand CM’s role in enhancing athletic performance.

## 1. Introduction

Citrulline is a non-protein, non-essential amino acid that has garnered significant interest in recent years for its potential to enhance exercise performance, primarily due to its role as an endogenous precursor to L-arginine, the main substrate of nitric oxide [1]. Citrulline is one of the most popular ergogenic ingredients included in pre-workout supplements, as found in a study investigating their use by both recreational and professional athletes [2]. Citrulline is commonly taken as citrulline malate (CM), a form in which malic acid is combined with L-citrulline in ratios ranging from 1:1 to 2:1 [3]. Systematic reviews and meta-analyses on citrulline supplementation generally highlight its potential benefits in delaying fatigue during high-intensity strength training [1], reducing muscle soreness, and improving post-exercise recovery [4]. However, as some studies have failed to demonstrate its benefits on overall performance [5,6,7], research on the performance benefits of citrulline remains inconclusive. Further studies are necessary to elucidate its effectiveness and determine the optimal conditions for its use.

The potential efficacy of CM in enhancing performance relies on three primary metabolic mechanisms: (1) improving blood and oxygen delivery during exercise via the L-arginine-NO pathway [8], (2) delaying fatigue and supporting oxidative metabolism by enhancing ammonia clearance through the urea cycle, thereby reducing blood lactate accumulation and facilitating the use of pyruvate for oxidative metabolism [9], and (3) potentially increasing adenosine triphosphate (ATP) turnover, as malate is involved in anaplerotic reactions within the TCA cycle, with its dehydrogenation into oxaloacetate being critical for sustained aerobic ATP production [10]. Given these proposed benefits, CM shows great potential in delaying fatigue and improving post-exercise recovery to enhance overall performance. To explore these benefits, numerous studies, both acute and chronic, have demonstrated its positive effects across various types of exercise, including endurance, high-intensity, and resistance training [4]. These benefits are particularly relevant for improving CrossFit^®^ performance, as CrossFit^®^ combines high-intensity training and fatiguing exercises where high amounts of lactate accumulation and increased ATP requirements are present [11]. Yet, to our knowledge, no study has been conducted on the effects of CM on CrossFit^®^ performance.

CrossFit^®^ is a rapidly growing high-intensity functional training program with affiliates in over 150 countries worldwide [12]. It aims to optimize physical competence across ten fitness domains, including cardiovascular endurance, strength, flexibility, and balance [13]. The intense and competitive nature of CrossFit, designed to enhance multidimensional fitness, likely contributes to its rising popularity and growing number of affiliates [14]. CrossFit^®^ workouts, known as “workouts of the day” (WODs), involve performing high-intensity exercises quickly, repetitively, and with minimal or no recovery time between sets [15]. A study comparing CrossFit (CF) to ACSM-based training found CF to be more intense, with participants describing it as “very hard” [16]. The training incorporates elements of gymnastics, weightlifting, and cardiovascular activities, emphasizing constantly varied functional movements to enhance overall fitness [13]. Due to its nature, CrossFit^®^‘s training paradigm often demands advanced level techniques performed at maximum intensity with minimal rest between sets [17]. This high-intensity approach, combined with an insufficient recovery time between high-volume loads and training sessions, can lead to early fatigue. The overload can result in increased oxidative stress, reduced resistance to repetitive exercise strain, a heightened perception of effort, and potentially unsafe movement execution [13]. Compared to ACSM-based training, CrossFit leads to higher fatigue, muscle soreness, swelling, and movement difficulties within 48 h post-workout. Workouts of the day (WODs) demand high physiological exertion, with heart rates reaching 54–98% HRmax, blood lactate levels of 6–15 mmol/L, and RPE scores of 8–9/10 [13]. Due to its demanding nature, CrossFit participants often seek solutions to enhance their performance and overall wellbeing. A study of 2576 CrossFit^®^ practitioners revealed a strong tendency towards supplement use to improve post-exercise recovery, with the majority (82.2%) using at least one ergogenic nutritional supplement. Pre-workout supplements were the most popular (20.7%) [18]. Given that most pre-workout supplements (70%) include CM [2], it is likely that CrossFit^®^ practitioners are using CM to enhance their performance. However, no studies have specifically investigated the impact of CM alone on CrossFit^®^ performance. Therefore, the primary purpose of this study is to examine the effects of CM as compared to the placebo on CrossFit^®^ workout performance. CM supplementation was hypothesized to improve performance as compared to the placebo. Secondary study purposes included examining effects of CM as compared to the placebo on (a) heart rate response, (b) time spent in heart rate intensity zones, and (c) post-exercise recovery time. For all three, CM was hypothesized to improve post-exercise recovery by increasing the time spent in higher intensity zones, reducing recovery time, and decreasing heart rate response.

## 2. Materials and Methods

### 2.1. Participants

A total of 21 healthy, recreationally active young men, aged 18–35 years, took part in this study (mean ± SD: body height 178 ± 5 cm, body weight 75.9 ± 10.4 kg, body fat percentage 12.7 ± 4.6%). Participants were recruited through study flyers and University social media channels. The inclusion criteria were the following: (1) having at least 3 months of CrossFit^®^ experience and having completed the “Cindy” CrossFit^®^ exercise. The study occurred during the 5-week preparation phase of the annual training program, with all participants following the Cindy protocol and completing five weekly sessions focused on strength, endurance, and power. A total of 11 volunteers were excluded based on the following criteria: (2) taking medication or dietary ergogenic aids within one year prior to the study, (3) failure to provide detailed nutrition and exercise logs, or (4) having any medical conditions that might affect performance. All participants were informed about the study procedures and provided written informed consent prior to participation. This study received approval from the Eötvös Loránd University Research Ethics Committee (2023/364) and was conducted in accordance with the Declaration of Helsinki.

### 2.2. Study Procedure

**Figure 1:**
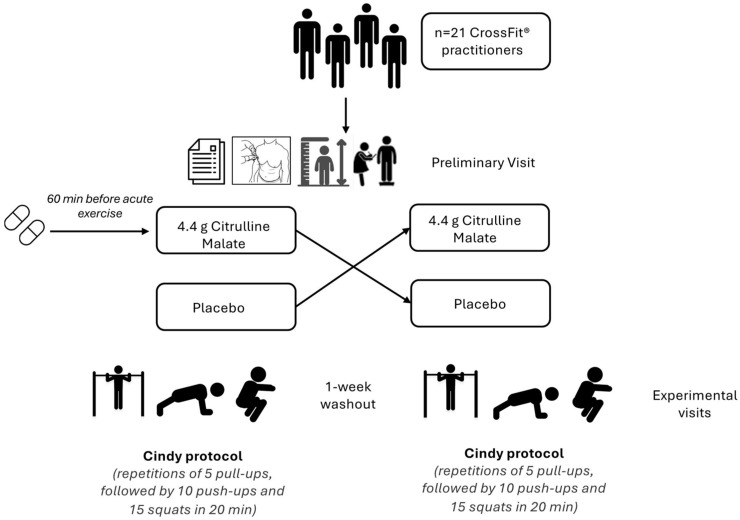
Study Flow Diagram.

In a randomized, double-blind, crossover study design, participants were first assigned to two groups at random using an online randomization tool, and a one-week washout period was implemented to eliminate any carryover effects (Figure 1). During the experimental visits, everything was set identically except the supplement administered. Both the CM and placebo capsules were identical in size, color, and appearance to prevent any indication of the supplements ingested. To ensure blinding, an independent investigator (pharmacist) handled the preparation and administration of all supplements, making sure that both the researchers and participants were unaware of the specific conditions. The researchers responsible for measuring outcomes were kept completely separate from the randomization process and did not know the allocation sequence at any point during the study or data analysis. They consumed either two capsules of CM (4.4 g total, with each capsule containing 2.2 g) (BioTechUSA Kft., Budapest, Hungary) or two placebo capsules (4.4 g total, with each capsule containing 2.2 g) 60 min before starting the Cindy workout protocol. The participants consumed a substantial amount of water (250–300 mL) with the capsules to support supplement absorption. The supplementation timing was determined according to recommendations from previous research on the effects of CM on exercise performance. Participants were instructed to avoid alcohol, caffeine, strenuous exercise, and nicotine for 24 h before coming to the laboratory. They were advised to maintain their regular diet, stay well hydrated, refrain from eating three hours before testing, and continue their usual training routine throughout the study. During the initial visit, a trained ISAK Level 1 anthropometrist gathered anthropometric measurements. These included height, measured with a stadiometer (Seca 217, Hamburg, Germany), and body weight, measured with a calibrated scale (InBody 720, Seoul, Republic of Korea), following ISAK measurement procedures. All participants completed a study questionnaire that included questions on their chronological age, years of training experience, and type of sport.

The “Cindy” workout was chosen due to its status as a standardized CrossFit^®^ routine, well documented in the literature. Participants completed as many rounds as possible within 20 min, adhering to CrossFit^®^ movement standards. Each round consisted of five pull-ups, ten push-ups, and fifteen air squats. The performance was measured by the total number of rounds completed within the 20 min timeframe. Certified CrossFit^®^ Level 1 or 2 evaluators verbally counted rounds and excluded repetitions that did not meet the movement standards, providing immediate feedback to ensure compliance. Each participant performed the workout individually, without any visible clock, timing device, or music. The exercise protocol was conducted indoors at a room temperature of 22 °C.

Heart rate (HR) measurements were taken before, during, and after the Cindy workout using a Polar Team Pro system (Polar Electro, v3.5.4, Kempele, Finland). The system includes a chest belt equipped with a sensor unit (Polar H7 Bluetooth 4.0 smart chest strap) that features built-in ECG electrodes, a 10 Hz integrated GPS, and a 200 Hz microelectromechanical system motion sensor. The Data collected by this device were transmitted to the Polar Beat software (v3.5.4). The Polar Team Pro software records heart rate (HR), tracks time spent in various intensity zones (seconds), measures distance covered, and monitors speeds (meters). Additionally, it calculates the load on the circulatory system, recovery time (hours), energy expenditure (Kcal), and heart rate variability (HRV), which is the variation in time intervals between consecutive heartbeats [19].

### 2.3. Statistical Analysis

An a priori power analysis was conducted for a repeated measures Analysis of Variance (ANOVA) to assess within-between interactions in a study examining the effects of acute CM supplementation on weightlifting exercise performance [20]. Assuming an anticipated effect size (ES) of 0.44, an alpha level of 0.05, two measurements, two groups, a correlation of 0.5 between measurements, a statistical power of 80%, and a nonsphericity correction epsilon of 1, the analysis determined that 14 participants were needed. However, we included 16 participants to account for potential dropouts, incomplete data, and to ensure the robustness of our findings. This power analysis was performed using G*Power (version 3.1.9.7; Düsseldorf, Germany).

Data analyses were performed using SPSS 29.0 statistical software (IBM, Armonk, NY, USA). All data are presented as mean and standard deviation (SD). Dependent variables were checked for normality using the Kolmogorov–Smirnov and Shapiro–Wilk tests before further analysis. The Wilcoxon sign rank test was used to compare the CM and placebo trials and to identify any potential learning effects between the first and second sessions. Percent changes in performance between sessions due to the learning effect were calculated with the formula: ((session 2–session 1)/session 1) × 100. Percent changes in repetitions due to the supplement were calculated using: ((carnitine—placebo)/placebo) × 100. A two-way repeated measures ANOVA (supplement × time) with partial eta-square effect size was used to analyze the CrossFit^®^ exercise performance following CM and placebo supplementation. Statistical significance was set at *p* < 0.05. The magnitude of differences between CM and placebo trials was evaluated using Cohen’s d, with effect sizes classified as follows: large (d > 0.8), moderate (d = 0.8–0.5), small (d = 0.5–0.2), and trivial (d < 0.2).

## 3. Results

The anthropometric characteristics of the participants are detailed in Table 1. The mean age of the participants was 22.2 ± 2.6 years, while the mean training experience (year) was 14.3 ± 3.8 years. The average body height was 178.3 ± 5.8 cm, and the mean body weight was 75.9 ± 10.4 kg. Regarding body composition, the participants had an average fat mass of 9.9 ± 4.8 kg, representing 12.7 ± 4.6% of their total body weight. The mean muscle mass was 37.7 ± 4.2 kg, which constituted 49.9 ± 2.6% of their body weight. Additionally, the mean lean body mass was 65.9 ± 7.0 kg, and the total body water averaged 49.6 ± 8.6 kg.

Table 2 illustrates the performance-related parameters of both conditions. In the CM group, a significant difference was observed between the time spent in P intensity zone 4 (M = 527 ± 395 s) and P intensity zone 5 (M = 194 ± 349 s) (F(1,20) = 6.762, *p* = 0.017, η^2^_p_ = 0.25), favoring zone 4.

Table 3 shows the total number of rounds completed during sessions, alongside the percentage changes in performance metrics across these sessions. There was a significant difference in the rounds completed during the first and second sessions (12.4 ± 5.3 vs. 14.9 ± 6.3 rounds, Z = −3.394, *p* < 0.001), demonstrating a learning effect. No significant difference was found for the rounds completed between supplement and placebo conditions (13.5 ± 5.2 vs. 13.8 ± 6.7 rounds, Z = −0.544, *p* = 0.587).

## 4. Discussion

This study evaluated the impact of acute CM supplementation on Cindy workout performance and recovery, as determined by heart rate response and post-exercise recovery time via HR monitor, in CrossFit^®^ practitioners. The main findings indicated that acute CM supplementation had no significant effect on exercise performance or recovery time. However, a significant learning effect was observed between the first and second sessions of “Cindy.” In addition, another aim of this study was to enhance the literature on CM supplementation and exercise performance by assessing its impact on training zones identified through HR data, thereby contributing to a more comprehensive understanding of its potential benefits in athletic performance. Results showed that the intensity of exercise was significantly lower in the CM trial (towards zone 4) compared to the placebo.

No significant performance improvement was detected between CM and placebo conditions in the present study. These findings align with some of the existing literature, suggesting that CM does not consistently provide ergogenic benefits during high-intensity exercise protocols [21,22,23]. An acute CM supplementation study at a dose of 12 g/day indicated no ergogenic benefit on time-to-exhaustion in well-trained individuals [21]. Another study evaluating the acute effect of CM supplementation (8 g taken 40 min before exercise) on upper-body resistance performance also showed that CM did not improve exercise performance in recreationally resistance-trained men [23]. Since CrossFit^®^ combines high-intensity workouts with functional multi-joint movements [13], it can be compared to other studies on high-intensity exercises. However, direct comparisons are challenging due to variations in protocols, exercise types, supplement doses and timing, citrulline/malate ratios, and participant demographics. Therefore, further research is needed to strengthen the evidence for CM as an ergogenic aid and to understand its mechanisms under different conditions of high-intensity exercise models.

It is important to note that a significant learning effect was observed between the first and second sessions. Similar results have been found in other CrossFit^®^ studies, with performance improvements of 16% [24] and 7.3% [25] within sessions. Crawford’s intervention showed physical fitness improvements ranging from 3.3% to 8.8%, whereas Stein et al. [25] did not find any performance benefits within trials. It was recommended to conduct at least two [26] to three [27] familiarization sessions when using unfamiliar protocols to minimize systematic error. To address this, although we did not conduct a formal familiarization session, we asked participants if they had previously completed the “Cindy” workout. Although the learning effect had a small effect size, a 22.9% improvement in CrossFit^®^ performance could be significant during competition. These findings suggest that the learning effect during CrossFit^®^ workouts may overshadow the effects of ergogenic aids, even among experienced CrossFit^®^ participants, where the learning effect is presumed to be minimal.

The present findings showed that although CM did not significantly increase CrossFit^®^ performance, as assessed by the number of rounds during each trial, time spent in zone 4 was significantly increased while time spent in zone 5 decreased in the CM condition compared to the placebo. A study by Kliszczewicz et al. [28] found that during the ‘Cindy’ benchmark WOD, CrossFit^®^ beginners maintained heart rates above 93% of their maximum, classifying the exercise as vigorous according to the American College of Sports Medicine [29]. Between 80 and 90% of HR max is classified as hard intensity, compared to above 90% of HR max, where athletes may enhance their ability to sustain high-speed endurance [30,31]. This increased time in zone 4 suggests that CM supplementation may be effective in improving both aerobic capacity and anaerobic threshold levels in CrossFit^®^ practitioners. Additionally, these findings support the notion that acute CM may have a small ergogenic effect on muscle endurance during high-intensity strength training, as demonstrated in a recent meta-analysis of eight studies [32]. Therefore, while CM may not directly boost CrossFit^®^ performance in terms of rounds completed, it appears to enhance the physiological factors that contribute to sustained high-intensity effort.

Considering the hypothetical benefits of CM in delaying fatigue, studies on CM often investigate its efficacy during or post-exercise recovery [20,21,33]. A meta-analysis on pre-exercise citrulline supplementation (either 3–6 g of L-citrulline or 6–12 g of citrulline malate) showed that citrulline supplementation decreased muscle soreness and the rating of perceived exertion (RPE), regardless of blood lactate modulation, in a sample of mostly trained individuals [4]. The included studies mainly determined its efficacy by measuring muscle soreness, blood lactate levels, and/or RPE using the BORG or OMNI-RPE scale. These findings are inconsistent with the results of the current study, as we did not find any significant difference in recovery time, determined by the HR monitor, after the Cindy workout in CrossFit^®^ practitioners. Additionally, few studies align with our results, showing no benefits of acute CM supplementation [7,34,35]. One possible reason for the inconsistency could be the different methodologies used to measure recovery. While previous studies primarily relied on either objective (via blood lactate levels or fatigue-related biomarkers) [5,6,21,33,36] or subjective measures such as muscle soreness and RPE [20,23], our study used heart rate monitoring to determine recovery time. The discrepancy might also be due to differences in the exercise protocols, sample populations, or the acute dose of CM supplementation in our study compared to others (4.4 g vs. 8 g CM, respectively). Although there is no certain recommended dose for CM supplementation, further research, including various recovery predictors and applying a higher CM dose in CrossFit^®^ practitioners, is needed to clarify these findings.

The strength of this study stems from its cross-over, placebo-controlled, double-blinded design, enabling participants to act as their own controls and accurately assess the effects of acute supplementation. We utilized the Cindy workout protocol, a well-established training regimen, to evaluate exercise performance in CrossFit^®^ practitioners. However, several limitations should be noted. We administered a dose of 4.4 g of CM (1:1 ratio), while the literature typically recommends a dose of 3–6 g of L-citrulline or 8 g of CM [4]. No familiarization sessions were conducted prior to the experimental protocol. Nevertheless, to mitigate this, we included only CrossFit^®^ athletes experienced with the Cindy protocol, suggesting the lack of familiarization sessions likely did not significantly impact the results. The experimental protocols were carried out with participants alone, without a visible clock or music, to maintain internal validity. However, CrossFit^®^ gyms usually feature music, numerous people, and a visible clock, which could influence real-life performance. Although a 24 h exercise restriction was prescribed to control for the potential impact of recent training sessions, we did not collect training logs during the experimental period. This could affect the results, as weekly training volume might influence performance due to potential overtraining or fatigue/delayed onset muscle soreness from other sessions. Lastly, we only included male CrossFit^®^ athletes, limiting the generalizability of the findings to female CrossFit^®^ athletes.

## 5. Conclusions

This study found that a 4.4 g dose of CM taken prior to the “Cindy” workout did not significantly enhance performance, as measured by the number of rounds completed, in recreational CrossFit^®^ athletes. Additionally, no significant improvements in post-exercise recovery time were observed. However, CM supplementation did result in increased time spent in the high-intensity zone 4, suggesting potential benefits for aerobic capacity and anaerobic threshold. These findings align with some of the existing literature, indicating that while CM may not directly improve performance metrics, it could enhance certain physiological factors. Further research is needed, with larger sample sizes, the inclusion of both genders, and an evaluation of both acute and chronic CM supplementation, to fully understand the potential benefits of CM in CrossFit^®^ athletes.

## Figures and Tables

**Table 1:** Anthropometric and body composition characteristics of participants.

	Mean ± SD (*n* = 21)
Age (year)	22.2 ± 2.6
Training Experience (year)	14.3 ± 3.8
Body Height (cm)	178.3 ± 5.8
Body Weight (kg)	75.9 ± 10.4
Fat Mass (kg)	9.9 ± 4.8
Fat Percentage (%)	12.7 ± 4.6
Lean Body Mass (kg)	65.9 ± 7.0
Total Body Water (kg)	49.6 ± 8.6

**Table 2:** Performance metrics for placebo (EA) and citrulline malate (CM) conditions in ‘CrossFit^®^’ ZCindy exercises.

	EA	CM	F	*p*	η^2^_p_
	Mean ± SD SD	Mean ± SD			
PO (bpm)	67.3 ± 23.0	72.8 ± 18.8	2.060	0.167	0.09
P_max_ (bpm)	183.8 ± 18.3	181.2 ± 11.5	0.433	0.518	0.02
P_mean_ (bpm)	148.1 ± 14.8	151 ± 16	2.032	0.169	0.09
P intensity zone (4) (s)	453 ± 334	527 ^a^ ± 395	2.217	0.152	0.10
P intensity zone (5) (s)	237 ± 344	194 ^b^ ± 349	1.102	0.306	0.05
Total distance (m)	482 ± 179	492 ± 178	0.231	0.636	0.01
Max. speed (km/h)	6.9 ± 0.7	7.1 ± 1.2	0.761	0.393	0.04
Mean speed (km/h)	1.21 ± 0.49	1.2 ± 0.5	1.079	0.311	0.05
Total load	55.9 ± 17.6	57.3 ± 16.6	0.668	0.423	0.03
Cardio load	42.6 ± 15.1	42.4 ± 14.2	0.005	0.945	0.00
Recovery time (h)	6.9 ± 4.7	6.6 ± 4.7	0.529	0.475	0.03
Calories (Kcal)	297 ± 60	297 ± 56	0.000	0.993	0.00
Mean R-R interval (ms)	415 ± 42	403 ± 45	3.071	0.095	0.13
HRV (RMSSD) (ms)	31.5 ± 21.0	25.9 ± 14.8	1.304	0.267	0.06

Abbreviation: PO: minimum pulse rate before exercise, Pmax: maximum pulse rate during exercise, P_mean_: mean pulse rate during exercise, P intensity zone 4: 80–90% of HRmax (152–172 bpm); P intensity zone 5: 90–100% of HR max (171–190 bpm), Rr_max_: Maximum time between consecutive heartbeats (beat-to-beat interval) recorded during the training session (milliseconds), Rr_mean_: the mean difference between HRmax and HRmin during each respiratory cycle (milliseconds), HRV(RMSSD): The mean square root of the difference in successive RR intervals. ^a,b^: paired *t*-test, *p* < 0.05, η^2^_p_ = 0.17.

**Table 3:** Percent changes in performance between different sessions, indicating a learning effect, and conditions specified by total rounds.

Subject ID	Session 1 Treatment	Session 1 Total Rounds	Session 2 Total Rounds	Percent Changes between Sessions	Percent Changes between Conditions
A01	CM	7.0	9.0	28.6	−22.2
A02	CM	6.0	5.0	−16.7	20.0
A03	CM	14.0	13.0	−7.1	7.7
A04	CM	9.0	9.0	0.0	0.0
A05	CM	11.0	10.0	10.0	−9.1
A06	CM	21.0	24.0	14.3	−12.5
A07	CM	21.0	24.0	14.3	−12.5
A08	CM	25.0	19.0	31.6	−24.0
A09	CM	16.0	21.0	31.3	−23.8
A10	CM	23.0	18.0	27.8	−21.7
A11	CM	9.0	11.0	22.2	−18.2
A12	CM	12.0	16.0	33.3	−25.0
A13	Placebo	25.0	23.0	8.7	8.7
A14	Placebo	6.0	15.0	150.0	150.0
A15	Placebo	11.0	7.0	57.1	57.1
A16	Placebo	10.0	12.0	20.0	20.0
A17	Placebo	11.0	8.0	37.5	37.5
A18	Placebo	9.0	7.0	−22.2	−22.2
A19	Placebo	15.0	12.0	25.0	25.0
A20	Placebo	15.0	12.0	25.0	25.0
A21	Placebo	11.0	10.0	−9.1	−9.1

## Data Availability

The data presented in this study can be obtained by contacting the corresponding author, subject to privacy and ethical considerations.
